# Evaluation of the osteoarthritis disease burden in China from 1990 to 2021: based on the Global Burden of Disease Study 2021

**DOI:** 10.3389/fpubh.2024.1478710

**Published:** 2024-11-15

**Authors:** Jiahui Liang, Yue Wang, Fei Yu, Guichun Jiang, Weiguo Zhang, Kang Tian

**Affiliations:** ^1^Department of Joint and Sports Medicine, First Affiliated Hospital of Dalian Medical University, Dalian, Liaoning, China; ^2^School of Graduates, Dalian Medical University, Dalian, Liaoning, China; ^3^The School of Medicine, Nankai University, Tianjin, China; ^4^Department of Nuclear Medicine, First Affiliated Hospital of Dalian Medical University, Dalian, Liaoning, China; ^5^Liaoning Cancer Hospital & Institute, Clinical Skills Training Center, Shenyang, China; ^6^Key Laboratory of Molecular Mechanism for Repair and Remodeling of Orthopaedic Diseases, Dalian, Liaoning, China

**Keywords:** Global Burden of Disease Study, osteoarthritis, incidence, prevalence, disability-adjusted life years, China

## Abstract

**Objective:**

This study aims to understand the current status and trend of the disease burden of osteoarthritis (OA) in people over 30 years old in China from 1990 to 2021 and identify the priority population groups, to provide reference data for the prevention and treatment of OA in China.

**Methods:**

The Global Burden of Disease Study 2021 was used to describe the incidence, prevalence, and disability-adjusted life years (DALYs) of OA in adult aged 30 years or older in China by sex and age groups, which was used to analyze the disease burden of OA from 1990 to 2021. The joinpoint regression model and age-period-cohort model were used to characterize the temporal trend.

**Results:**

In 2021, the number of OA prevalence in China was about 152.85 million, and the number of incidences was about 11.65 million. The age-standardized prevalence rate (ASPR), age-standardized incidence rate (ASIR) and age-standardized DALYs rate (ASDR) of OA in China are lower than those in developed countries such as Korea, the United States of America and Japan, but higher than those in India. Knee osteoarthritis had the highest ASPR and ASDR. The highest incidence rate was 50–54 years, and the highest prevalence and DALYs rate were in the age group of patients over 95 years old. The incidence rate of women in all age groups is higher than that of men. From 1990 to 2021, the ASIR, ASPR and ASDR of OA in China roughly showed an increasing trend year by year. The age-period-cohort analysis showed that the peak age groups for longitudinal age incidence of OA in China from 1992 to 2021 were 50–54 years and 80–84 years. We found that the OA incidence was highest in the period 2017–2021.

**Conclusion:**

The disease burden of OA in those over 30 years old in China from 1990 to 2021 will become more and more serious, and the target population for primary prevention is the female population under 50 years old. The development of a scientific and effective comprehensive prevention and treatment program for OA is imminent.

## 1 Introduction

Osteoarthritis (OA) is a common chronic and disabling condition that mainly affects middle-aged and older adults. It mainly affects the hands, hips, knees and other joints that are subject to high levels of stress and activity. As the disease progresses, joint activity may be limited or even deformed in severe cases (1, 2). It dramatically changes the patient’s lifestyle and, in advanced stages, can lead to joint replacement, placing a heavy burden on the patient, the family and the social healthcare system (3). As the population is aging at an accelerating rate, 2021–2030 has been designated as the Decade of Healthy Aging by the United Nations General Assembly. The rapid change in China’s demographic structure during the previous four decades suggests that the burden of aging in China will continue to increase (4). Some studies showed that the number of OA prevalence in China in 2019 was 10,681,311 and there were 527,811,871 OA patients worldwide. The disability-adjusted life years (DALYs) of OA patients in China are about 4.72 million person-years, accounting for 24.93% of the global DALYs due to OA (5, 6). Although OA is common in older patients, it is also inevitable in younger patients. Studies have shown that the disease burden of early-onset OA is also increasing in patients younger than 55 years (7). The above evidence shows that OA is not only a heavy burden to the Chinese healthcare system, but also a huge blow to humanity worldwide. Scientists have explored the pathogenic mechanisms and treatment methods of OA, but most of them are still in the animal experimental stage, and there are no effective measures to reverse OA lesions. Therefore, it is particularly important to implement comprehensive preventive measures (8, 9).

In recent years, research on the burden of diseases of OA has been concentrated on global epidemiologic studies, with few thorough studies on the disease burden of OA in China. One study predicted the trend of OA in the next few years and suggested that the age-standardized rate would decrease in women and increase in men (10). There are also studies predicting that the age-standardized prevalence rate (ASPR), age-standardized incidence rate (ASIR) and age-standardized DALYs rate (ASDR) of OA will decrease year by year between 2020 and 2035 (5). However, the author hypothesizes that the disease burden associated with OA will rise over time.

The Global Burden of Disease Study 2021 (GBD 2021) was recently updated on May 16, 2024. This article uses the GBD 2021 to analyze the prevalence, incidence, and DLAYs of OA in China from 1990 to 2021 to assess the impact of OA disability on people’s quality of life and to provide a scientific foundation for the development of OA prevention and treatment policies in China. This will help readers clearly understand the disease burden and evolving trends associated with OA in China.

## 2 Methods

### 2.1 Overview and source of data

The data in this article are from the GBD 2021, a study conducted by the Institute for Health Metrics and Evaluation (IHME) at the University of Washington, which regularly publishes the Global Health Data Exchange (GHDx), and can be downloaded from the website.^1^ The database offers a thorough evaluation of total and cause-specific incidence, prevalence, mortality and disability for 371 diseases corresponding to 88 risk factors in 204 countries and territories for the period 1990–2021, using standardized methods of analysis based on age and gender.

The retrieval strategy for the GBD 2021 is as follows: “GBD estimate”: cause of death or injury; “Measure”: DALYs, incidence, prevalence; “Metric”: number, percentage, rate; “Cause”: osteoarthritis, osteoarthritis knee, osteoarthritis hip, osteoarthritis hand and osteoarthritis other; “Location”: China, Global, Africa, America, Asia, Europe, India, Japan, Korea, United States of America; “Age”: all ages, age-standardized, 30 years to >95 years; “Sex”: both, female, male; “Year”: from 1992 to 2021.

We adopted a publicly accessible database for secondary analyses in our work, which was exempt from ethical constraints because it did not involve human subjects or animals.

### 2.2 Case definitions

The GBD 2021 classifies OA based on the International Classification of Diseases, 10th edition (ICD 10), code B.11.2 Osteoarthritis, which is categorized as osteoarthritis of the hand, hip, knee and other forms. OA cases are defined as OA associated with clinical symptoms and confirmed by radiologic imaging in Kellgren–Lawrence (K–L) II–IV. K–L II is characterized by the presence of well-defined osteophytes in the joints and suspected joint space stenosis; K–L III is characterized by the presence of multiple osteophytes and significant joint space stenosis, and K–L IV is characterized by the presence of a large number of osteophytes and significant joint deformity (11, 12).

DALYs, an epidemiologic indicator that assesses the duration of disability in a population of deceased and survivors, is composed of two components: years of life lost due to premature mortality (YLLs) and years lived with disability (YLDs), i.e., DALYs = YLLs + YLDs (9). However, the death of OA was not directly assigned in the GBD 2021, so YLLs = 0 and DALYs = YLDs. Therefore, DALYs are directly used in this study to analyze the impact of OA. In addition, no statistics on OA-related information on people under 30 years old were kept in the GBD 2021, so the target population of this study is people over 30 years old.

### 2.3 Descriptive analysis

In this study, the data on OA in China were sorted and analyzed using Excel 2021 and R Studio software. The burden of OA in China was described using the incidence, prevalence, DALYs, ASIR, ASPR and ASDR in the GBD 2021. The GBD study reports age-standardized estimates and mean estimates with a 95% uncertainty interval (UI). Its estimations are quantified more than 1,000 times, and the 95% UI is calculated using the 25th and 975th values from the ordered 1,000 estimates (13).

### 2.4 Joinpoint regression model

The Joinpoint software is an epidemiological statistics software that enables trend analysis. You can download the latest version from the website of the National Cancer Institute^2^. The principle of the joinpoint regression (JPR) model is to start with a minimum number of join points, test all of them for statistical significance and finally join the points with similar trends into a smooth straight line (14). The JPR model can be used to analyze the temporal characteristics of diseases in terms of incidence, prevalence and DALYs (15). Therefore, the JPR model was used in this study to evaluate the average annual percentage change (AAPC) and annual percentage change (APC) of ASPR, ASIR and ASDR of OA in China. APC is used to assess the change over a period of time, while AAPC is a comprehensive measure of the overall change over time. APC > 0 indicates a year-on-year increase, otherwise a decrease. JPR models are divided into linear and non-linear models (16). In this study, non-linear regression analysis was used, and a statistically significant difference was defined as a *p*-value of less than 0.05.

### 2.5 Age-period-cohort analysis

The age-period-cohort (APC) model is widely used in epidemiology in areas such as chronic non-communicable diseases and cancer (17). This model can decompose the factors of period, birth cohort and age in the epidemiological data, and more accurately estimate the effects of the three independent factors on the incidence or mortality of the disease. It can also examine how the disease has changed over time in a long-term trend. In the APC analysis, this study analyzed the data on the incidence and DALYs rate of osteoarthritis in China by age group, which were compiled every 5 years from 1992 to 2021. APC models were constructed using the APC Web Tool and age-specific rates ratio (RR) were assessed for each period and cohort (18). A statistically significant difference was defined as a *p*-value of less than 0.05.

### 2.6 Statistical analysis

In this study, the software R Studio (version 4.2.1) was used for statistical description and plotting. Joinpoint software (version 5.2.0) was used to calculate APC, APCC, and 95% CI and to construct relevant models to analyze the trend changes in OA. In addition, we used the APC Web Tool to construct an APC model to assess the age-specific rate ratio (RR) for each period and cohort. A statistically significant difference was defined as a *p*-value of <0.05.

## 3 Results

### 3.1 Disease burden of OA and different types of OA in China in 1990 and 2021

Between 1990 and 2021, the number of incidences, prevalence and DALYs of OA in China increased from 4.65 million (95% UI: 4.08–5.21), 53.35 million (95% UI: 46.60–59.69) and 1.83 million (95% UI: 0.88–3.68) person-years to 11.65 million (95% UI: 10.21–13.11), 152.85 million (95% UI: 134.66–170.84) and 5.32 million (95% UI: 2.54–10.68) person-years, representing relative increases of 150.54, 186.50 and 190.71% (Supplementary Table S1). The ASIR, ASPR and ASDR increased from 487.11 per 100,000 (95% UI: 428.13–543.75), 6,148.92 per 100,000 (95% UI: 5,417.29–6,855.85) and 210.61 per 100,000 (95% UI: 101.91–423.86) to 554.61 per 100,000 (95% UI: 486.85–619.54), 7,030.66 per 100,000 (95% UI: 6,211.20–7,831.69) and 244.79 per 100,000 (95% UI: 117.30–491.91), the relative increase was 13.86, 14.34, and 16.23%, respectively (Table 1).

**Table 1:** Age-standardized rate of prevalence, incidence, DALYs in 1990 and 2021 for osteoarthritis to China.

Measure	1990 ASR per 100,000 (95% UI)			2021 ASR per 100,000 (95% UI)		
	Both	Male	Female	Both	Male	Female
Prevalence	6148.92 (5417.29, 6855.85)	4922.14 (4337.36, 5518.70)	7334.28 (6454.23, 8156.89)	7030.66 (6211.20, 7831.69)	5678.50 (5004.04, 6364.73)	8322.14 (7380.75, 9242.63)
Incidence	487.11 (428.13, 543.75)	389.19 (341.93, 436.26)	589.07 (516.89, 656.91)	554.61 (486.85, 619.54)	445.31 (390.68, 498.52)	664.05 (584.15, 742.68)
DALYs	210.61 (101.91, 423.86)	168.91 (81.49, 341.08)	250.86 (121.26, 503.47)	244.79 (117.30, 491.91)	197.25 (94.41, 397.28)	290.18 (139.28, 582.90)

ASR, age-standardized rate; DALYs, disability-adjusted life years; UI, uncertainty interval.

In 2021, the ASPR of the four types of OA in China was, in descending order, osteoarthritis knee (5,016.52 per 100,000), osteoarthritis hand (1,603.85 per 100,000), osteoarthritis other (710.15 per 100,000) and osteoarthritis hip (260.10 per 100,000). Compared to 1990, the ASIR for knee OA, hand OA, hip OA and other OA increased by 7.54, 56.80, 30.5 and 5.58%, respectively. In 2021, the ASDR for knee OA was 162.44 per 100,000, accounting for 66.36% of all types of OA (Table 2). We find that while the rise in hand OA incidence is the most obvious, knee OA has the most serious negative impact on the population.

**Table 2:** All age cases prevalence, incidence and DALYs number and age-standardized rate of osteoarthritis in China in 1990 and 2021.

Diseases	Year	Prevalence		Incidence		DALYs	
		Number (million)	Rate (per100,000)	Number (million)	Rate (per100,000)	Number (million)	Rate (per100,000)
Osteoarthritis	1990	53.35	6,148.92	4.65	487.11	1.83	210.61
	2021	152.85	7,030.66	11.65	554.61	5.33	244.79
Osteoarthritis knee	1990	41.04	4,667.29	3.65	377.93	1.34	151.24
	2021	109.58	5,016.52	8.51	406.42	3.55	162.44
Osteoarthritis hip	1990	1.72	202.34	0.10	10.62	0.06	6.52
	2021	5.47	260.10	0.29	13.86	0.18	8.35
Osteoarthritis hand	1990	7.99	988.51	0.54	59.12	0.26	31.57
	2021	34.42	1,603.85	1.96	92.70	1.10	51.26
Osteoarthritis other	1990	5.42	663.47	0.37	39.43	0.18	21.27
	2021	15.35	710.15	0.89	41.63	0.49	22.75

DALYs, disability-adjusted life years.

### 3.2 Disease burden of OA in different regions of the world in 2021

**Figure 1:**
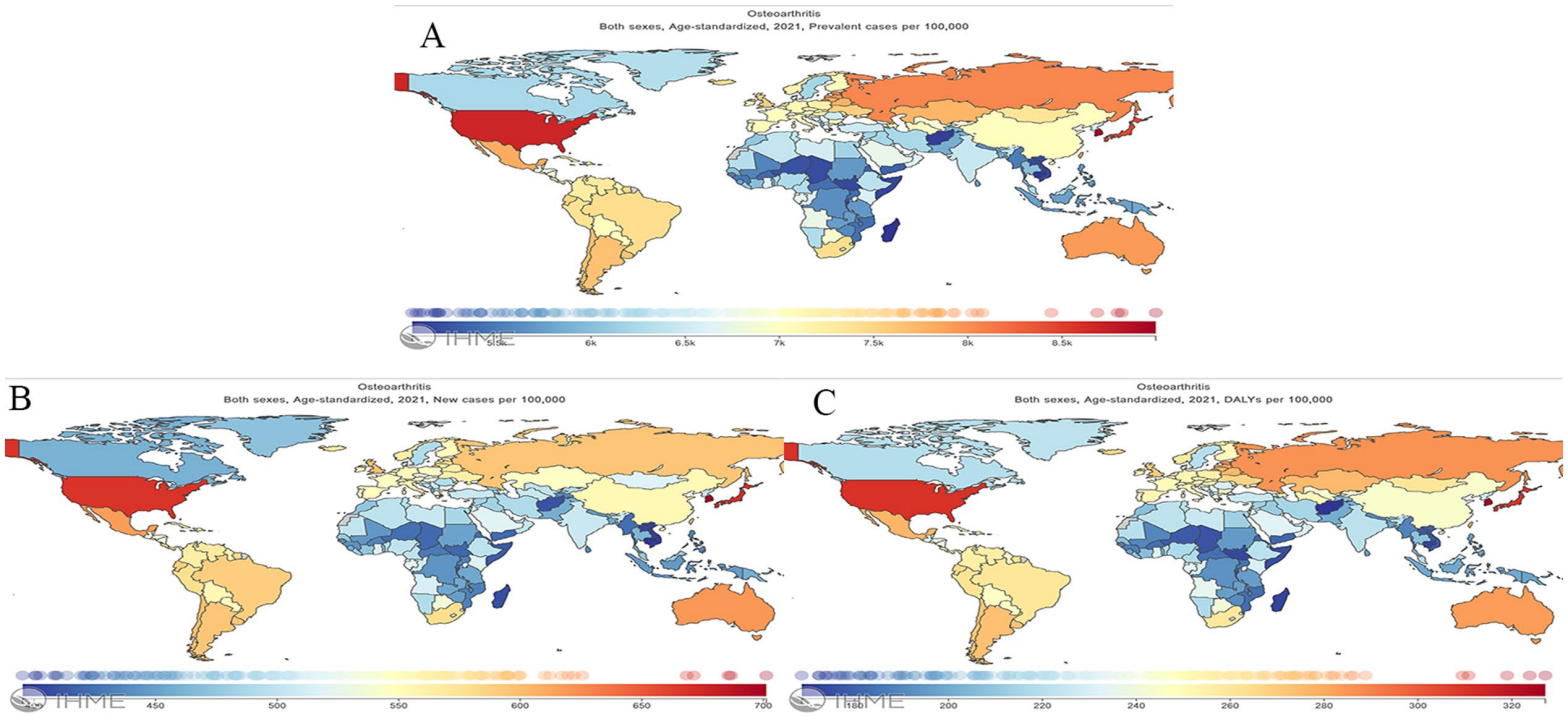
Geographical distribution of age-standardized rate of Prevalence, Incidence and DALY for osteoarthritis worldwide in 2021. **(A)** Prevalence; **(B)** incidence; **(C)** DALYs; DALYs, disability-adjusted life years.

In 2021, the ASPR of OA in China is 7030.66 per 100,000, which is lower than developed countries such as Korea (8997.39 per 100,000), the United States of America (USA) (8686.57 per 100,000) and Japan (8442.68 per 100,000) and higher than India (6450.10 per 100,000). The ASIR for OA in China is 554.61 per 100,000, which is lower than developed countries such as Korea (701.23 per 100,000), the USA (668.49 per 100,000) and Japan (671.40 per 100,000) and higher than India (505.00 per 100,000). Similarly, ASDR for OA in China is 244.79 per 100,000, which is lower than developed countries such as Korea (327.14 per 100,000), the USA (310.78 per 100,000) and Japan (309.47 per 100,000) and higher than India (221.21 per 100,000). The geographical distribution of ASIR, ASPR and ASDR of OA in different regions of the world is shown in Figure 1 (the global geographical visualization presented herein was derived through analysis utilizing the interactive visualization interface of the IHME website). From Figure 1, we can compare the disease burden of OA between China and other countries in the world. China is a developing country, so we just compare China with other developing countries in Asia such as India, with developed countries such as Japan and Korea, and at the same time with the developed country with heavy disease burden, the United States of America, to highlight the disease burden of OA in China. In addition, the disease burden in China is higher than in the global, Africa and Asia and lower than in America and Europe, compared to the world and the four world regions (Supplementary Table S2).

### 3.3 Disease burden by sex and different age groups in China in 2021

**Figure 2:**
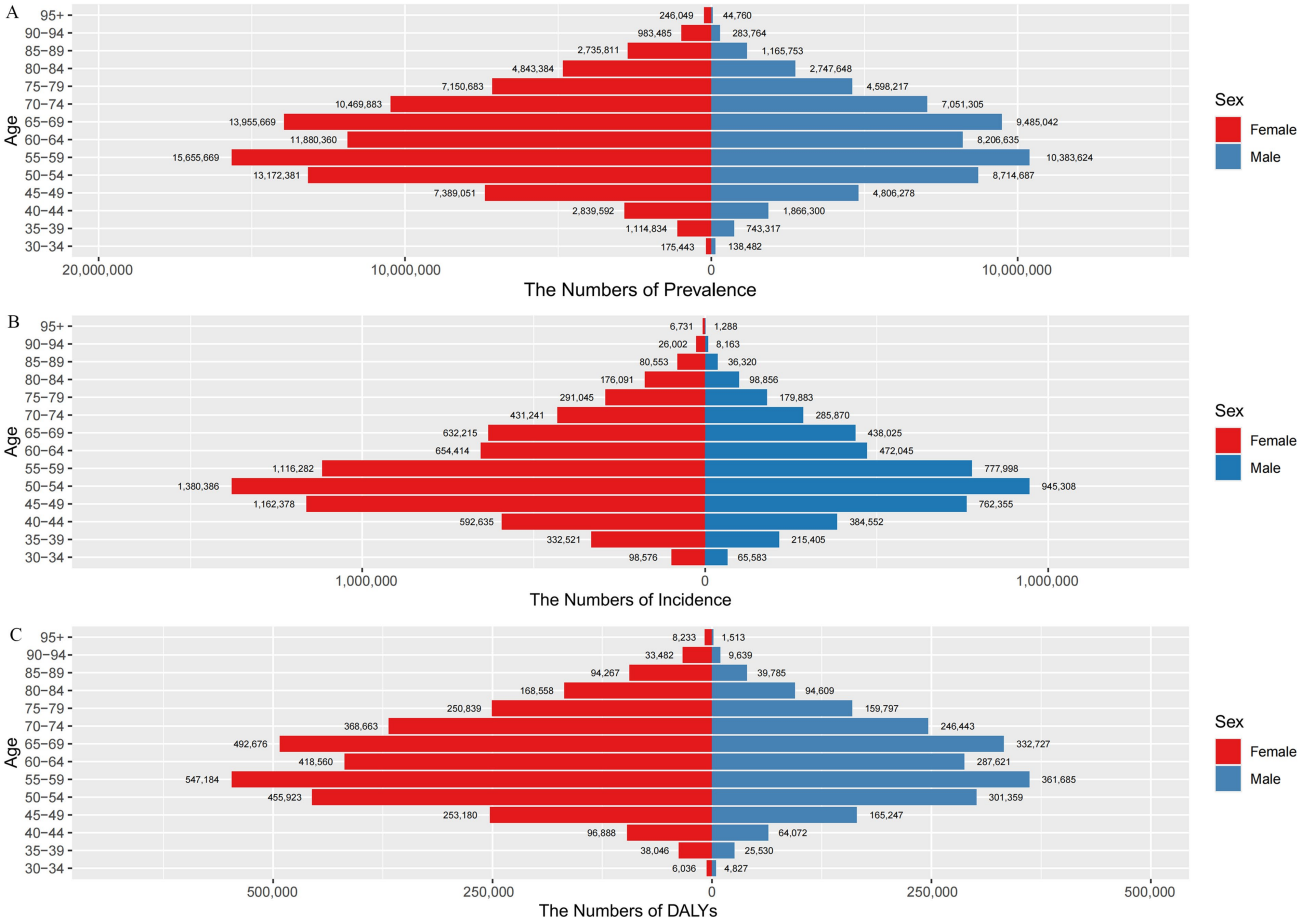
Number of cases and incidence, prevalence and DALYs of osteoarthritis according to sex and age group in China in 2021. **(A)** Prevalence; **(B)** incidence; **(C)** DALYs; DALYs, disability-adjusted life years.

**Figure 3:**
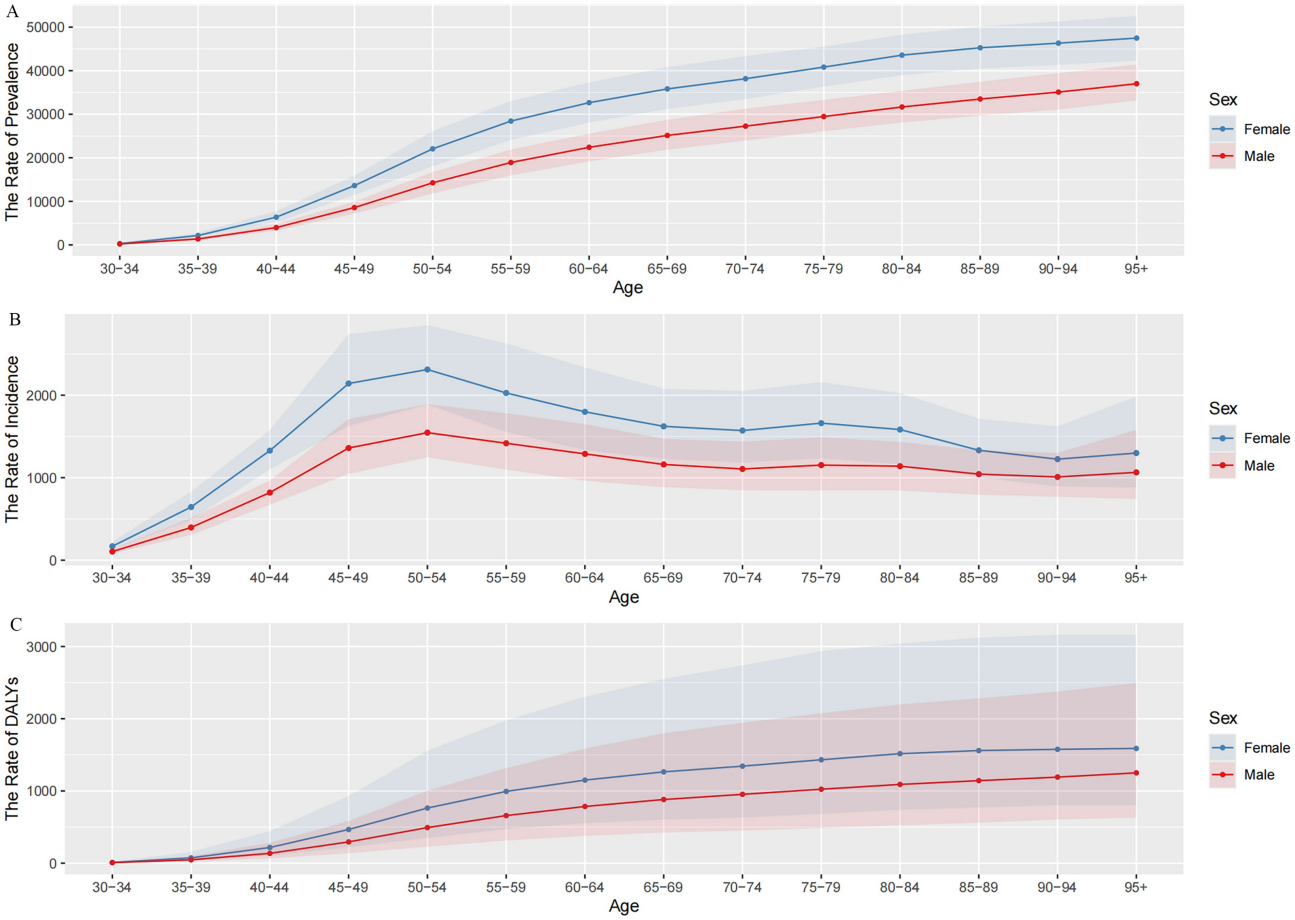
Trends in osteoarthritis prevalence, incidence, and DALYs rates by sex and age group in China in 2021. **(A)** The rate of prevalence; **(B)** the rate of incidence; **(C)** the rate of DALYs; DALYs, disability-adjusted life years.

From the analysis of GBD 2021 data, the prevalence number of OA in China in 2021 is about 152.85 million (95% UI: 134.66–170.84), accounting for 11.21% of the total prevalence in China and 25.18% of the global prevalence of OA. The number of women with OA (92.61 million) is significantly higher than that of men (60.24 million) (Figure 2A). Furthermore, in every age group, women have a higher prevalence rate than men. With increasing age, the population’s prevalence rate steadily rose, with women over 95 having the greatest prevalence rate at 47,485.55 per 100,000 (95% UI: 42,295.72–52,582.97), which is 1.28 times higher than that in men in the same age group (Figure 3A).

The number of OA incidence in China has exceeded 10 million every year since 2014, and the number of OA incidence cases was 11.65 million in 2021 (95% UI: 10.21–13.11), accounting for 0.26% of the total incidence in China and 24.99% of the global incidence of OA, with more cases occurring in women (6.98 million) than men (4.67 million) (Figure 2B). The incidence rate of OA in China increased rapidly from 135.50 per 100,000 (95% UI: 103.82–175.95) in the 30–34 age group and peaked at 1,924.30 per 100,000 (95% UI: 1,560.47–2,371.26) in the 50–54 age group, then gradually declined (Figure 3B). In 2021, the ASIR of OA is 1.49 times higher in women (664.05 per 100,000) than in men (445.31 per 100,000). In addition, the incidence rate is higher in women than in men in all age groups, namely about 1.5 times higher than in men in the same age group. The highest incidence rate is found in women aged 50–54 years (2,311.51 per 100,000).

In 2021, the number of DALYs due to OA in China amounted to 5.33 million person-years, accounting for 1.32% of DALYs from all causes in China and 25.00% of DALYs due to OA in the world. The overall number of DALYs was significantly higher for women (4,653,500 person-years) than for men (2,877,100 person-years) (Figure 2C). The rate of DALYs for OA in China shows an increasing trend with age, and women in the 95+ age group have the highest rate of DALYs (1,588.88 per 100,000), which is 1.27 times higher than that of men (1,250.80 per 100,000) in the same age group (Figure 3C).

### 3.4 Disease burden by sex and different years in China

**Figure 4:**
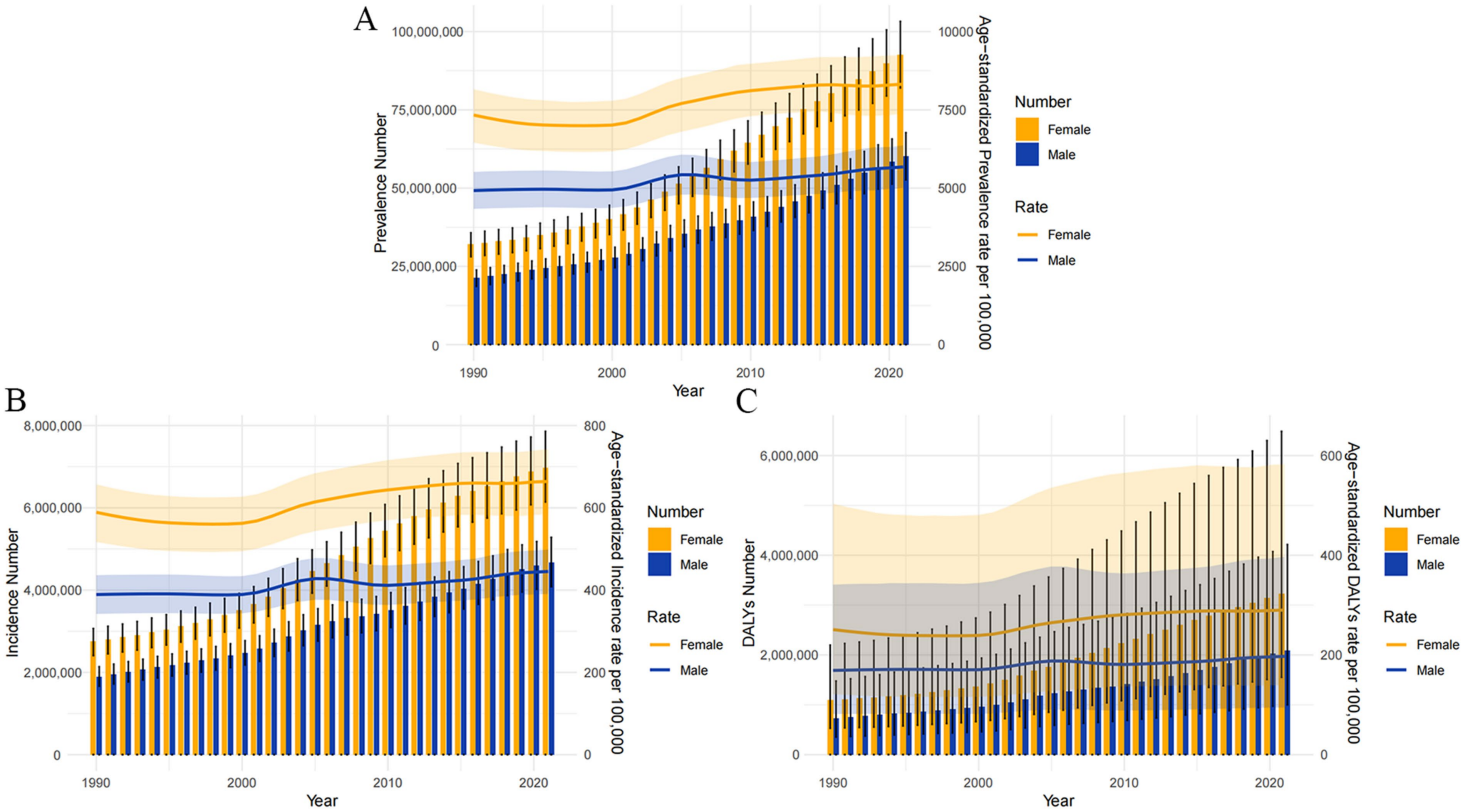
The Number of osteoarthritis prevalence, incidence and DALYs (the bar graph with left *Y*-axis) and age-standardized prevalence, incidence and DALYs rate (per 100,000) by sex (the line graph with right *Y*-axis) from 1990 to 2021. **(A)** Prevalence; **(B)** incidence; **(C)** DALYs; DALYs, disability-adjusted life years.

From 1990 to 2021, the ASIR, ASPR and ASDR of OA in China were higher in women than in men (Figure 4). In 2021, the ASIR of OA in men and women in China was 445.31 per 100,000 (95% UI: 390.68–498.52) and 664.05 per 100,000 (95% UI: 584.15–742.68), respectively, and the ASPR was 5,678.50 per 100,000 (95% UI: 5,004.04–6,364.73) and 8,322.14 per 100,000 (95% UI: 7,380.75–9,242.63), and the ASDR were 197.25 per 100,000 (95% UI: 94.41–397.28) and 290.18 per 100,000 (95% UI: 139.28–582.90), respectively (Table 1). The three assessment indicators of ASIR, ASPR, and ASDR showed a significantly different distribution between men and women according to gender distribution, and OA patients were predominantly women.

### 3.5 The joinpoint regression analysis of the ASPR, ASIR, and ASDR of OA in China from 1990 to 2021

**Figure 5:**
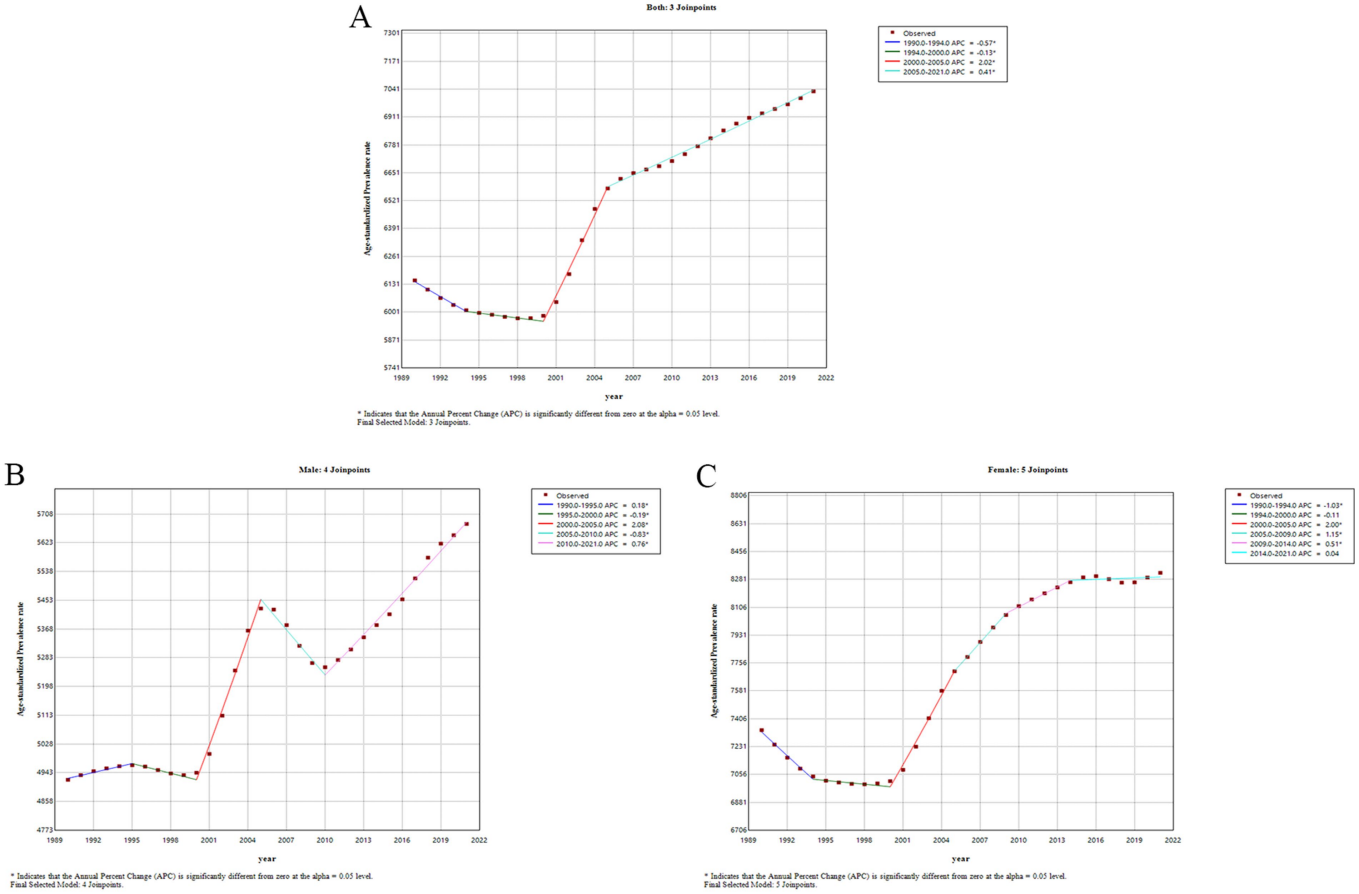
Results of the joinpoint regression models for trend analysis of age-standardized prevalence rates of osteoarthritis in China from 1990 to 2021. **(A)** Both; **(B)** male; **(C)** female.

Table 3, Figure 5, and Supplementary Figures S1, S2 display the outcomes of the joinpoint regression analysis. For all patients in China, the trend for ASPR of OA decreased from 1990 to 2021 and then increased (Figure 5A). From 1990 to 2000, there was a minor decrease, and from 2000 to 2005, there was a notable increase (APC: 2.02; 95% CI: 1.88–2.17) and a slow increase in ASPR from 2005 to 2021 (APC: 0.41; 95% CI: 0.40–0.43). The ASPR of all people increased by an average of 44% per year (AAPC = 0.44; 95% CI: 0.41–0.47, *p* < 0.05).

**Table 3:** Results of the joinpoint regression models for trend analysis of age-standardized prevalence, incidence and DALYs rates of osteoarthritis in China from 1990 to 2021.

Trend	Prevalence		Incidence		DALYs	
	Time interval	APC (95% CI)	Time interval	APC (95% CI)	Time interval	APC (95% CI)
Both						
Trend 1	1990–1994	−0.57 (−0.71, −0.43)*	1990–1994	−0.55 (−0.66, −0.44)*	1990–1994	−0.58 (−0.70, −0.45)*
Trend 2	1994–2000	−0.13 (−0.23, −0.03)*	1994–2000	−0.14 (−0.22, −0.06)*	1994–2000	−0.13 (−0.22, −0.04)*
Trend 3	2000–2005	2.02 (1.88, 2.17)*	2000–2005	2.01 (1.89, 2.12)*	2000–2005	2.15 (2.02, 2.28)*
Trend 4	2005–2021	0.41 (0.40, 0.43)*	2005–2009	0.18 (0.01, 0.36)*	2005–2021	0.48 (0.46, 0.49)*
Trend 5			2009–2021	0.47 (0.45, 0.49) *		
AAPC	1990–2021	0.44 (0.41, 0.47)*	1990–2021	0.43 (0.39–0.46)*	1990–2021	0.49 (0.46–0.52)*
Male						
Trend 1	1990–1995	0.18 (0.05, 0.31)*	1990–2000	−0.06 (−0.10, −0.02)*	1990–1995	0.26 (0.10, 0.42)*
Trend 2	1995–2000	−0.19 (−0.37, −0.01)*	2000–2005	2.04 (1.88, 2.21)*	1995–2000	−0.19 (−0.41, 0.04)
Trend 3	2000–2005	2.08 (1.89, 2.26)*	2005–2010	−0.90 (−1.06, −0.74)*	2000–2005	2.16 (1.93, 2.39)*
Trend 4	2005–2010	−0.83 (−1.00, −0.66)*	2010–2015	0.62 (0.46, 0.78)*	2005–2010	−0.90 (−1.12, −0.68)*
Trend 5	2010–2021	0.76 (0.72, 0.79)*	2015–2019	1.07 (0.82, 1.33)*	2010–2014	0.67 (0.31, 1.02)*
Trend 6			2019–2021	0.37 (−0.15, 0.89)	2014–2021	0.96 (0.86, 1.05)*
AAPC	1990–2021	0.46 (0.41, 0.51)*	1990–2021	0.42 (0.36–0.48)*	1990–2021	0.51 (0.43–0.59)*
Female						
Trend 1	1990–1994	−1.03 (−1.20, −0.86)*	1990–1994	−1.04 (−1.17, −0.90)*	1990–1994	−1.09 (−1.23, −0.95)*
Trend 2	1994–2000	−0.11 (−0.23, 0.01)	1994–2000	−0.15 (−0.24, −0.06)*	1994–2000	−0.12 (−0.22, −0.02)*
Trend 3	2000–2005	2.00 (1.83, 2.18)*	2000–2005	1.92 (1.78, 2.05)*	2000–2005	2.16 (2.02, 2.31)*
Trend 4	2005–2009	1.15 (0.89, 1.41)*	2005–2009	0.99 (0.79, 1.20)*	2005–2009	1.33 (1.10, 1.56)*
Trend 5	2009–2014	0.51 (0.35, 0.67)*	2009–2014	0.56 (0.43, 0.68)*	2009–2014	0.56 (0.42, 0.71)*
Trend 6	2014–2021	0.04 (−0.03, 0.11)	2014–2021	0.11 (0.06, 0.17)*	2014–2021	0.12 (0.06, 0.18)*
AAPC	1990–2021	0.40 (0.35, 0.46)*	1990–2021	0.39 (0.34–0.43)*	1990–2021	0.47 (0.42–0.52)*

DALYs, disability-adjusted life years; APC, annual percent change; AAPC, average annual percent change; CI, confidential interval. *P < 0.05.

A separate analysis of gender revealed that ASPR increased in waves in male patients (Figure 5B). The prevalence rate gradually increased from 1990 to 1995 (APC: 0.18; 95% CI: 0.05–0.31) and then slowly decreased from 1995 to 2000 (APC: −0.19; 95% CI: −0.37, −0.01), followed by a dramatic increase in prevalence from 2000 to 2005 (APC: 2.08; 95% CI: 1.89–2.26). From 2005 to 2010, it gradually decreased (APC: −0.83; 95% CI: −1.00, −0.66), which was followed by a steady increase in prevalence from 2010 to 2021 (APC: 0.76; 95% CI: 0.72–0.79). The percentage of ASPR increased by an average of 46% per year (AAPC = 0.46; 95% CI: 0.41–0.51, *p* < 0.05). In female patients (Figure 5C), ASPR decreased slowly from 1990 to 2000 and then gradually increased, with the most significant increase from 2000 to 2005 (APC: 2.00; 95% CI: 1.83–2.18). The percentage of ASPR increased on average by 40% per year (AAPC = 0.40; 95% CI: 0.35–0.46, *p* < 0.05). Compared to women, the increasing trend in the ASPR of OA was more pronounced in men.

The temporal trends of ASIR and ASDR were similar to those of ASPR, as shown in Supplementary Figures S1, S2. The ASIR of OA in the total population showed an upward trend from 1990 to 2021 (AAPC = 0.43; 95% CI: 0.39–0.46, *p* < 0.05). There was also an increasing trend in ASDR in the total population with OA from 1990 to 2021 (0.39–0.46) (AAPC = 0.49; 95% CI: 0.46–0.52, *p* < 0.05).

### 3.6 Age-period-cohort analysis

#### 3.6.1 Age effect for incidence and DALYs rate of OA in China from 1992 to 2021

**Figure 6:**
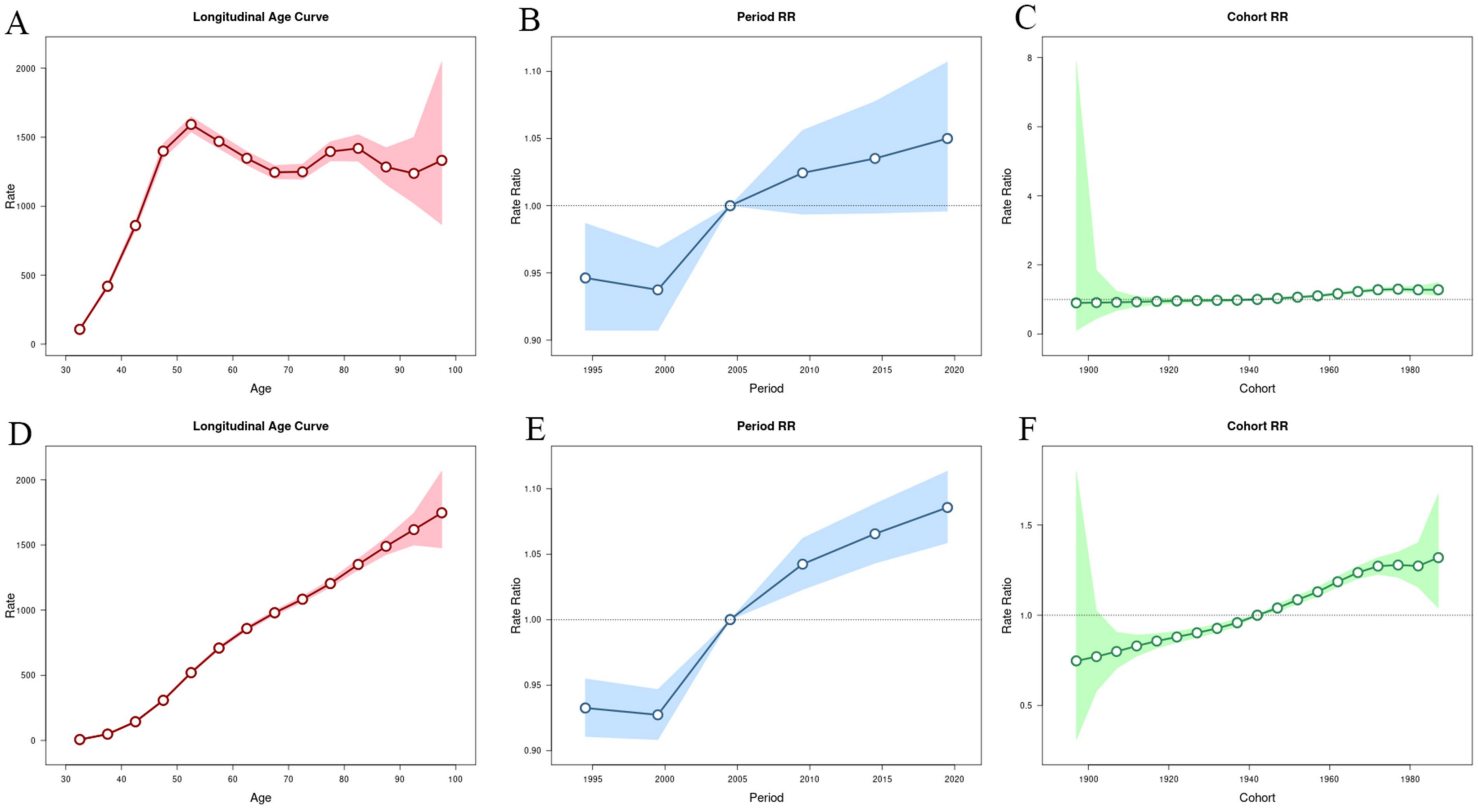
Age–period–cohort models for incidence and DALYs rates of osteoarthritis in China from 1992 to 2021. **(A,D)** Age effect for incidence and DALYs rate. **(B,E)** Period effect for incidence and DALYs rate. **(C,F)** Cohort effect for incidence and DALYs rate. DALYs: disability-adjusted life years.

The longitudinal age curve of the incidence of OA in China from 1992 to 2021 shows a general trend that initially increases and then slowly decreases with fluctuations, with a total of two peak age groups (Figure 6A). The first interval of incidence increase ranged from 107.50 per 100,000 (95% CI: 99.90–115.68) at the age of 30–34 years to 1,592.44 per 100,000 (95% CI: 1,538.07–1,648.73) at the age of 50–54 years. The second range of incidence increased from 1,245.47 per 100,000 (95% CI: 1,197.50–1,295.36) in 65–69 years to 1,419.07 per 100,000 (95% CI: 1,326.21–1,518.43) in 80–84 years. On the other hand, the DALYs rate of osteoarthritis in China increased with increasing age (Figure 6D).

#### 3.6.2 Period effect for incidence and DALYs rate of OA in China from 1992 to 2021

In China, the incidence and DALY rate ratios (RR) have gradually increased over time. The control group was from 2002 to 2006 (RR = 1.00), while the lowest period RR was from 1997 to 2001 (incidence RR = 0.94, 95% CI: 0.91–0.97), (DALYs RR = 0.93, 95% CI: 0.91–0.96). The period RR was highest from 2017 to 2021 (incidence RR = 1.05, 95% CI: 1.00–1.11), (DALYs RR = 1.09, 95% CI: 1.06–1.11). As shown in Figures 6B,E.

#### 3.6.3 Cohort effect for incidence and DALYs rate of OA in China from 1992 to 2021

In China, the RR of incidence and DALYs of OA increased with the transition of birth cohorts. We take the birth cohort from 1940 to 1944 as the reference value (RR = 1.00), the highest cohort RR of incidence was found in the birth cohort from 1975 to 1979 (RR = 1.30, 95% CI: 1.23–1.37), and the highest cohort RR of DALYs was found in the birth cohort after 1985–1989 (RR = 1.32, 95% CI: 1.04–1.67), as shown in Figures 6C,F.

## 4 Discussion

From 1990 to 2021, the rate of incidence, prevalence and DALYs of OA in China showed an increasing trend year by year. In 2021, the prevalence number of OA reached 152.85 million, accounting for 11.21% of the total number of domestic diseases of all causes and 25.18% of the global number of OA. The number of DALYs caused by OA in China was 5.33 million person-years, accounting for 1.32% of the DALYs caused by OA in China and 25.00% of the DALYs caused by OA in the world. Since 2014, the annual incidence number of OA in China has exceeded 10 million, accounting for 24.99% of the global incidence of OA. The above data clearly show that with the increasing aging trend of the population, considering the unique circumstances of China, it is imperative to develop a comprehensive OA prevention and control program. In this study, we investigated the global disease burden of OA and compared the Chinese data with the global average in different regions. The results show that the disease burden of OA is closely related to economic development and is greater in developed countries than in developing countries, which is consistent with the results of previous studies (6, 19). In addition, we compared China with other Asian countries and found that the disease burden caused by OA in China is much higher than in India and lower than in Japan and Korea, which is consistent with the conclusion of Hunter et al. (20). Genetic and ethnic variables might be the cause of this discrepancy. The results of the study show that knee osteoarthritis has the highest prevalence and DALYs rates, indicating that the disease burden of knee osteoarthritis is the highest. This supports the findings of earlier research (21, 22) and shows that China still needs to prevent and manage osteoarthritis in the knee.

A study by Hu et al. (13), based on the Global Burden of Disease Study 2019 (GBD 2019), characterized the burden of osteoarthritis in China from 1990 to 2019. However, their research primarily focused on joinpoint regression analysis and model predictions, omitting the disease burden of various types of osteoarthritis and a global comparative analysis. In addition to this, our study incorporates age-period-cohort analysis and provides corresponding visualizations for comparative analysis, enhancing reader comprehension. Most importantly, our research utilizes an updated database, which includes data from the last 2 years and revises the definition of osteoarthritis, offering insights into the most current burden of the disease in China. Compared to GBD 2019, our research reveals a continued increase in the burden of OA in China over the past 2 years. Our data presents a more compelling argument than Hu’s model, as it directly provides the most recent indicators of OA burden for the year 2021, serving as a valuable resource for scholars. Similarly, we identified the at-risk demographic for OA as middle-aged and old women, which is a crucial criterion for informing our preventive strategies.

The results of the study showed that the disease burden of OA was higher in women of all ages than in men of the same age, which is close to the results of a meta-analysis of publications on the epidemiology of OA in middle-aged and old Chinese people published by researchers in China between 2000 and 2018 (23). This conclusion indicates the key population for the prevention and control of OA, which is consistent with the recommended standards of the latest edition of the guidelines for the diagnosis and treatment of OA in China (24). Several studies have investigated the pathogenesis of OA and find that gender differences in OA are related to estrogen (25). Because estrogen protects articular cartilage and can limit the expression of phosphorylated epidermal growth factor receptor (p-EGFR) in the surface cartilage of the knee joint, it has a regulatory influence on osteoarthritis (26). Moreover, one of the most significant risk factors for OA is age (27). In this study, the incidence in the 50–54 age group was the highest, followed by a slow decline with increasing age, which we hypothesize may be related to changes in estrogen levels during menopause (25).

From the perspective of the burden of disease indicators, the prevalence, incidence and DALYs rate of OA increase with age, suggesting that the burden of disease caused by OA increases with age. As OA is a disabling but non-fatal condition, the results indicate that the incidence of OA peaks in the 50–54 age group, whereas the prevalence of OA and the DALYs rate peak in the 90+ age group (28). This is consistent with the results of Hu et al. (13) and Li et al. (6). However, it is interesting to note that our results are in contrast to those of Tang et al. (29). According to their findings, the prevalence of OA in the knee increases with age until the age of 70, at which point it levels off. This could be related to the different databases used and the fact that not every type of osteoarthritis was thoroughly investigated in this study, all of which are factors that could lead to different conclusions. The rate of DALYs in each age group of Chinese OA in 2021 was higher than the rate of DALYs in the previous age group. The increase in the prevalence of OA and the rate of DALYs was most pronounced in the 40–49 age group, suggesting that primary prevention should focus on women under the age of 50. The progression of OA should be prevented or slowed down using different methods depending on the characteristics of different population groups (30). Here are some recommendations for different age groups, 0–10 years: Monitoring and treatment of hip dysplasia; 10–15 years: Prevention of CAM deformities during growth plate closure; 15–45 years: Prevention of sports-related knee injuries and muscle weakness; 45–60 years: Prevention of obesity and inactivity; 60–80 years: prevention of age-related sarcopenia; In order to achieve early identification and diagnosis, OA patients should be encouraged to seek medical care in the early stage and their health education should be enhanced. Developing individualized treatment plans can help patients recover, reduce disability and lessen the burden of disease that OA places on individuals, families and society.

The joinpoint analysis showed that in China, the ASPR, ASIR and ASDR showed a decreasing trend from 1990 to 2000, and interestingly began to increase after 2000, with the fastest rate of increase from 2000 to 2005, which was roughly consistent with previous studies. There could be several reasons for this trend: the rapid economic growth after the economic reform and the tremendous progress in healthcare (31). The increasing income inequality in China, the rising life expectancy (32), the high body mass index (33, 34) and the aging of the population (35).

Using age-period-cohort models, we analyzed long-term trends in OA incidence and DALYs rates to give a theoretical foundation for the development of OA prevention and treatment strategies (36). The results on age effects showed that the highest incidence of OA occurred in the 50 to 54yearsold age group and gradually decreased with age thereafter. In contrast, the DALYs rate of OA increased with age. This suggests that the occurrence of osteoarthritis in middle age appears to be inevitable (37), and the older we get, the greater the burden of disability becomes. The incidence and progression of OA can be reduced by the interventions mentioned earlier, which are most effective when implemented in the early stages of the disease (38). The period effect refers to the fact that improvements in public health strategies, health education tools and medical levels have an impact on changes in the incidence and DALYs rate of osteoarthritis over a period of time (39). The results of the period effect study showed that the risks of both OA incidence rate and DALYs rate were low in the period before 2005 and then gradually increased. This could be related to comprehensive health education (40) and improved medical standards (41, 42). As living standards have improved and unhealthy habits have proliferated, the risk of OA has gradually increased. The cohort effect describes how a population’s birth age affects its exposure to different social, ecological, and environmental influences. The results of the cohort effect in this study showed that the risk of both incidence and DALYs rate increased with increasing birth years. This suggests that we should strengthen the prevention of OA to reduce the disease burden of OA.

In conclusion, the current situation of the disease burden of OA in China from 1990 to 2021 is still very serious, and China should invest lots of time and effort in the prevention and treatment of OA. Considering the differences in age and gender, middle-aged and older women should pay more attention to the treatment and prevention of OA. We recommend that prevention should be personalized according to the characteristics of different groups of people and that osteoarthritis should be prevented or controlled by reducing body weight, increasing exercise and avoiding joint injuries. First, overweight and obesity should be avoided to reduce stress on the knee and hip joints (43). Secondly, regular moderate physical activities such as swimming and walking are needed to strengthen the muscles around the joints and improve joint stability (44). Finally, it is important to avoid high-intensity activities and joint injuries and minimize wear and tear on articular cartilage by climbing fewer stairs and hills. Secondary and tertiary preventive measures, on the other hand, include advertising, education and training in populations and regions with a high incidence of OA, increasing health education about OA and encouraging patients in the early stages to take the initiative and see a doctor to enable early detection, diagnosis and treatment. China attaches great importance to the prevention of OA and has carried out a series of special initiatives and published the Guidelines for the Diagnosis and Treatment of Osteoarthritis in China, which guides the prevention and treatment of OA (45). The American College of Rheumatology (ACR) and the Arthritis Foundation have joined forces to develop evidence-based guidelines for the comprehensive management of OA, with a focus on prevention strategies for OA (46). At the international level, the World Health Organization (WHO) also emphasizes the prevention and treatment of osteoarthritis and suggests strategies to reduce symptoms through exercise and a healthy diet.

This paper reviews the current status of the main indicators of the disease burden of OA in China and the trend of changes over the past 30 years, which provides some scientific insights for the development of prevention and control programs. However, this study also has its limitations: first, the data of GBD 2021 mainly come from administrative epidemiological surveillance, registrations, censuses, disease registrations, etc., and the corresponding indicators are calculated according to mathematical modeling based on them, which are not the statistics of large-scale surveys strictly targeting OA, so there may be some discrepancy with the real situation; second, the study mainly focuses on China and does not analyze data from other countries. Third, our article does not analyze the risk factors for OA because the GBD database only provides one risk factor for high body mass index (BMI), and our subsequent study will explore all the risk factors for OA thoroughly. Therefore, our conclusions of the research are regional. Since the GBD 2021 does not provide statistical results broken down by provinces, the differences in disease burden across regions need to be further investigated.

## 5 Conclusion

Osteoarthritis is a major public health challenge. With the increasing aging of the population, the disease burden of OA in people over 30 years old in China will become more and more serious between 1990 and 2021, and in future, this trend will continue. The target population for primary prevention is the female population under 50 years old, and the development of a scientific and effective comprehensive prevention and treatment program for OA is imminent.

## Data Availability

Publicly available datasets were analyzed in this study. This data can be found: https://www.healthdata.org/research-analysis/gbd.

## Footnotes

^1^ https://www.healthdata.org/research-analysis/gbd

^2^ https://surveillance.cancer.gov/joinpoint/download

## Supplementary material

SUPPLEMENTARY FIGURE S1 Results of the joinpoint regression models for trend analysis of age-standardized incidence rates of osteoarthritis in China from 1990 to 2021. **(A)** Both; **(B)** male; **(C)** female.

SUPPLEMENTARY FIGURE S2 Results of the joinpoint regression models for trend analysis of age-standardized DALYs rates of osteoarthritis in China from 1990 to 2021. **(A)** Both; **(B)** male; **(C)** female; DALYs, disability-adjusted life years.
