# Supplementary material for: Evaluation of the osteoarthritis disease burden in China from 1990 to 2021: based on the Global Burden of Disease Study 2021
Source: Front Public Health. 2024 Nov 15;12:1478710. doi: 10.3389/fpubh.2024.1478710 (PMC11604587; doi:10.3389/fpubh.2024.1478710)
Supplement: Supplementary file 1 [file Table_1.DOCX]

| Measure | 1990  Number×10^6^ (95% UI) | | | 2021  Number×10^6^ (95% UI) | | |
| --- | --- | --- | --- | --- | --- | --- |
|  | Both | Male | Female | Both | Male | Female |
| Prevalence | 53.35  (46.60, 59.69) | 21.30  (18.63, 23.87) | 32.06  (28.08, 35.75) | 152.85  (134.66,170.84) | 60.24  (52.65,67.73) | 92.61  (81.98,103.27) |
| Incidence | 4.65  (4.08, 5.21) | 1.90  (1.66, 2.14) | 2.75  (2.41, 3.07) | 11.65  (10.21, 13.11) | 4.67  (4.08, 5.28) | 6.98  (6.14, 7.86) |
| DALYs | 1.83  (0.88, 3.68) | 0.73  (0.35, 1.48) | 1.10  (0.53, 2.20) | 5.33  (2.54,10.68) | 2.09  (1.00, 4.22) | 3.23  (1.55, 6.49) |

**Supplementary Table 1** All age cases Prevalence, Incidence, DALYs rates in 1990 and 2021 for osteoarthritis to China

DALYs: disability-adjusted life years; UI: uncertainty interval;

**Supplementary Table 2** The number and Age-standardized rate of Prevalence, Incidence, DALYs in 2021 for osteoarthritis to different regions in the world

| Regions | Prevalence | | Incidence | | DALYs | |
| --- | --- | --- | --- | --- | --- | --- |
|  | Number  (million) | ASR  (per100,000) | Number  (million) | ASR  (per100,000) | Number  (million) | ASR  (per100,000) |
| Global | 606.99 | 6967.29 | 46.63 | 535.00 | 21.30 | 244.50 |
| Africa | 42.50 | 6123.73 | 3.96 | 481.31 | 1.46 | 211.40 |
| America | 104.49 | 7886.64 | 7.69 | 610.01 | 3.72 | 279.92 |
| Asia | 351.10 | 6758.62 | 27.68 | 521.52 | 12.25 | 235.75 |
| Europe | 107.83 | 7234.82 | 7.24 | 553.72 | 3.84 | 256.89 |
| Korea | 8.44 | 8997.39 | 0.62 | 701.23 | 0.31 | 327.14 |
| USA | 47.58 | 8686.57 | 3.19 | 668.49 | 1.71 | 310.78 |
| Japan | 25.38 | 8442.68 | 1.51 | 671.40 | 0.94 | 309.47 |
| China | 152.85 | 7030.66 | 11.65 | 554.61 | 5.33 | 244.79 |
| India | 79.21 | 6450.10 | 6.70 | 505.00 | 2.72 | 221.21 |

ASR: Age-standardized rate; USA: the United States of America; DALYs: disability-adjusted life years
