# Supplementary figures and images for: Evaluation of the osteoarthritis disease burden in China from 1990 to 2021: based on the Global Burden of Disease Study 2021
Source: Front Public Health. 2024 Nov 15;12:1478710. doi: 10.3389/fpubh.2024.1478710 (PMC11604587; doi:10.3389/fpubh.2024.1478710)

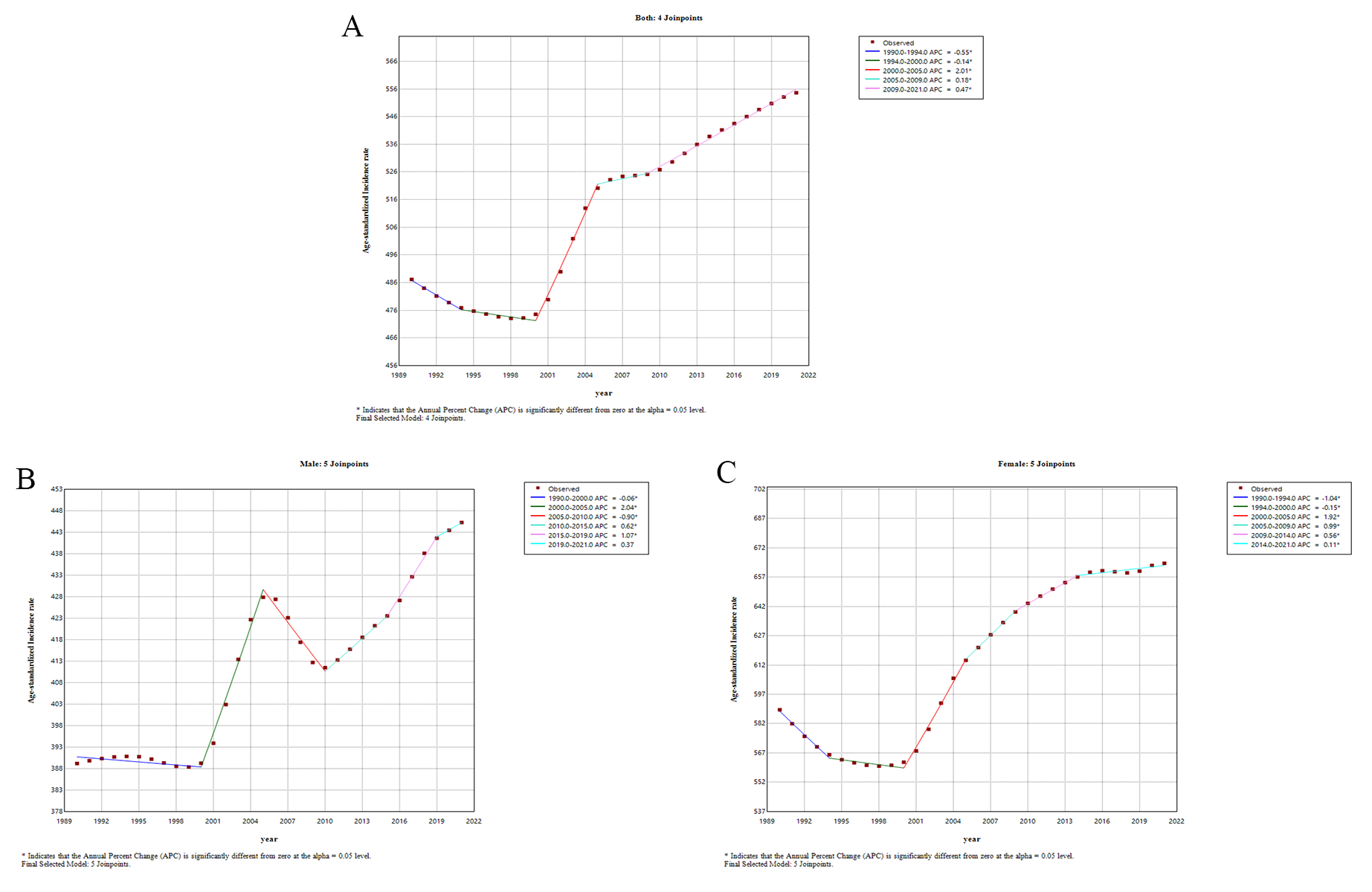

Supplement: SUPPLEMENTARY FIGURE S1 — Results of the joinpoint regression models for trend analysis of age-standardized incidence rates of osteoarthritis in China from 1990 to 2021. (A) Both; (B) male; (C) female. [file Image_1.TIF]

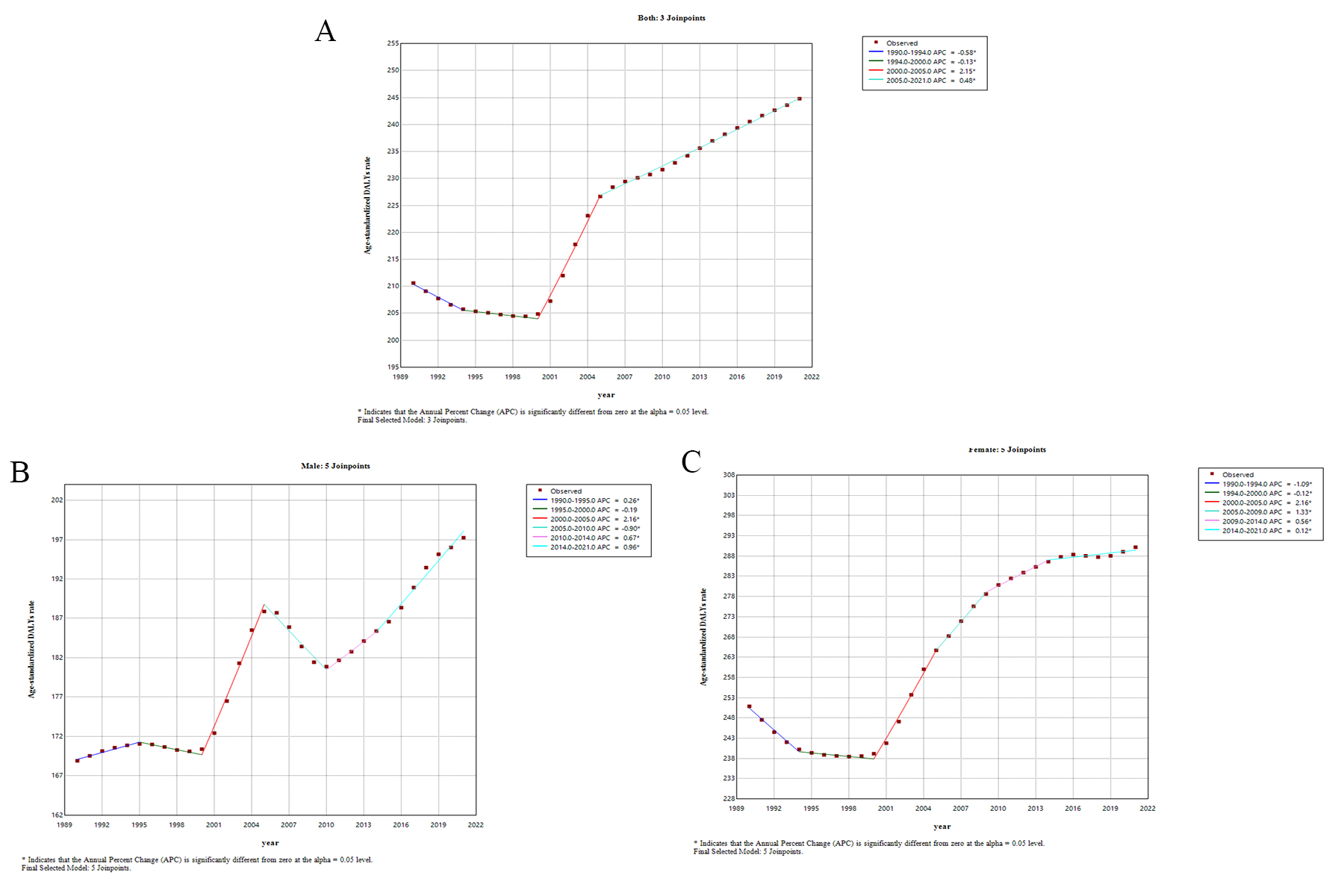

Supplement: SUPPLEMENTARY FIGURE S2 — Results of the joinpoint regression models for trend analysis of age-standardized DALYs rates of osteoarthritis in China from 1990 to 2021. (A) Both; (B) male; (C) female; DALYs, disability-adjusted life years. [file Image_2.TIF]
